# Integrated comparative metabolite profiling via NMR and GC–MS analyses for tongkat ali (*Eurycoma* *longifolia*) fingerprinting and quality control analysis

**DOI:** 10.1038/s41598-023-28551-x

**Published:** 2023-02-13

**Authors:** Ahmed Serag, Ahmed Zayed, Ahmed Mediani, Mohamed A. Farag

**Affiliations:** 1 Pharmaceutical Analytical Chemistry Department, Faculty of Pharmacy, Al-Azhar University, Cairo, 11751 Egypt; 2 Pharmacognosy Department, College of Pharmacy, Tanta University, Elguish Street (Medical Campus), Tanta, 31527 Egypt; 3 Institute of Systems Biology (INBIOSIS), Universiti Kebangsaan Malaysia (UKM), 43600 Bangi, Selangor Malaysia; 4 Pharmacognosy Department, College of Pharmacy, Cairo University, P.B. 11562, Kasr el Aini St., Cairo, Egypt

**Keywords:** Analytical chemistry, Mass spectrometry, NMR spectroscopy

## Abstract

Tongkat ali commonly known as Malaysian Ginseng (*Eurycoma* *longifolia*) is a herbal root worldwide available in nutraceuticals, either as a crude powder or capsules blended with other herbal products. Herein, a multiplexed metabolomics approach based on nuclear magnetic resonance (NMR) and solid-phase microextraction combined with gas chromatography–mass spectrometry (SPME–GC–MS) was applied for authentic tongkat ali extract vs some commercial products quality control analysis. NMR metabolite fingerprinting identified 15 major metabolites mostly ascribed to sugars, organic and fatty acids in addition to quassinoids and cinnamates. Following that, multivariate analysis as the non-supervised principal component analysis (PCA) and supervised orthogonal partial least squares-discriminant analysis (OPLS-DA) were applied revealing that differences were related to fatty acids and 13,21-dihydroeurycomanone being more enriched in authentic root. SPME–GC–MS aroma profiling led to the identification of 59 volatiles belonging mainly to alcohols, aldehydes/furans and sesquiterpene hydrocarbons. Results revealed that aroma of commercial products showed relatively different profiles being rich in vanillin, maltol, and methyl octanoate. Whereas *E*-cinnamaldehyde, *endo*-borneol, terpinen-4-ol, and benzaldehyde were more associated to the authentic product. The present study shed the light for the potential of metabolomics in authentication and standardization of tongkat ali and identification of its true flavor composition.

## Introduction

Recent advances in biological and analytical sciences applied for traditional herbal medicines as well as innovations in omics can undoubtedly play a leading role in the quality control and validation of commercial herbal products. Particularly, tongkat ali (Malaysian Ginseng) is the roots of *Eurycoma* *longifolia* tree (family Simaroubaceae) that is native to Southeast Asian countries, but it is widely consumed by millions of people all over the world, competing with Panax Ginseng (Chinese Ginseng)^1,2^. It is mostly grown in Malaysia for its therapeutic benefits to preserve wild plants^3^. Despite of its bitter taste, it has been consumed as coffee and tea additives based on its well reported effect in natural wellness and unique bioactive contents^4^. Moreover, folk medicine has documented the use of its roots extract for sexual dysfunction, and many other health disorders^1,2,5^. However, more accessible formulas are marketed nowadays either as a raw crude powder or capsules blended with other herbs^2^.

The aroma profile of tongkat ali roots as a main predictor of their hot beverage flavor, as well as impact of heating/roasting on their chemical compositions have been characterized in preliminary research based on soxhlet and conventional steam extraction methods which revealed few number of volatiles^6^. Nevertheless, solid phase microextraction (SPME) is an ultimate novel, simple, solvent-free, and sensitive approach for detecting volatiles at low temperatures that can be used to evaluate the true aroma of in *E.* *longifolia* roots^7,8^. Compared to steam distillation for aroma analysis, there are numerous advantages for non-exhaustive SPME technique compared to exhaustive extraction resulting in more representative information of volatiles composition^9^. SPME presents an optimum sample pretreatment process that eliminates any compounds that are incompatible with the instrument such as matrix compounds^10^. Thus, SPME presents a potential tool for VOCs analysis in the roots of tongkat ali especially considering its medium aroma. Coupled to SPME; gas chromatography with mass spectrometry (GC–MS) is the most suited for identification of aroma components^11,12^.

Asides from tongkat ali aroma components contributing to its flavor, root encompasses a wide range of non-volatile bioactive chemicals, including phenolics, triterpenes, alkaloids, and squalene derivatives^13,14^. One of the best platforms that can characterize such chemicals is NMR with numerous advantages of being high-throughput, highly reproducible, non-destructive, and universal with minimal sample preparation steps^15^. In addition, the NMR signal intensities are directly proportional to the corresponding real molar levels of the detected metabolites, which makes NMR an absolute quantitative method without any need for calibration curves of individual analytes. Besides, the comparison of the overall metabolic composition can be performed for different specimens using a data matrix produced from ^1^H NMR spectra aided by multivariate models^15–17^.

Hence, metabolomics research that employs NMR and MS as complimentary approaches may provide significant insights into tongkat ali root metabolome. The current study applies a complementary approach of NMR and GC–MS coupled with chemometric analyses for untargeted metabolite fingerprinting of *E.* *longifolia* roots extracts in comparison with some of its commercial products. The aim of this study was further to use NMR finger printing to standardize *E.* *longifolia* extract by quantifying its major components for future incorporation in quality control of nutraceuticals.

## Materials and methods

### Plant material and commercial preparations

Root specimen of cultivated *E.* *longifolia* was obtained from the Ethno Resources Sdn, Sungai Buloh. Malaysia. The plant material was authenticated by Dr. Idder Boubaker, Botanist of Plant Taxonomy at the Laboratory of microbiology and plant biology, University Abdulhamid Ibn Badis, Mostaganem, Algeria. In the laboratory's herbarium, voucher specimens (LMPB0017-2021) were kept. All procedures were conducted in conformity with the pertinent rules and regulations. Three commercial *E.* *longifolia* products, viz*.* Nu-prep LELAKI^®^ (each capsule contains 100 mg *E.* *longifolia* extract), Naturelle^®^ (each capsule contains 100 mg *E.* *longifolia* extract) and Long jack plus^®^ (each capsule contains 425 mg *E.* *longifolia,* 50 mg *Nigella* *sativa* and 25 mg *Cassia* *angustifolia* extracts) were obtained from retail stores in Kuala Lumpur Malaysia.

### Chemicals and reagents

Hexamethyldisiloxane (HMDS) and methanol-d4 (CD_3_OD) were purchased for NMR measurement as an internal standard from Deutero GmbH (Kastellaun, Germany). In addition, divinylbenzene-carboxen-polydimethylsiloxane (DVB-CAR-PDMS) and polydimethylsiloxane (PDMS) fibers provided in 1 cm long and 50/30 µm, from Supelco (Bellefonte, PA, USA), were used for SPME aroma sampling.

### Extraction procedure and samples preparation for NMR analysis

Samples extraction were performed following^18^. Briefly, 15 mg of the plant extract and the commercial preparations (according to the labels) were homogenized with 6 mL of MeOH using a Turrax mixer (11,000 rpm) for five 20 s periods. Extracts were vortexed vigorously to remove plant debris for 1–2 min and centrifuged at 3000×*g* for 30 min. Four mL of the root extracts and commercial products were dried under a stream of nitrogen, then resuspended with 700 µL CD_3_OD containing HMDS (0.94 mM). Afterwards, centrifugation at 13,000×*g* for 1 min, and finally the supernatant was transferred to a 5 mm NMR tube and subjected to NMR analysis. To account for biological variability, three biological replicates for each group were extracted and analyzed under identical conditions.

### NMR analysis

Agilent VNMRS 600 NMR spectrometer was used to record all NMR spectra operating at a ^1^H NMR frequency of 599.83 MHz and combined with implemented in Varian VNMRJ 2.2C spectrometer software. For ^1^H NMR experiments, the instrument’s parameters were adjusted to a relaxation delay = 17.95 s; pulse angle = 90°, pulse width (pw) = 6.25 μs and number of transients = 120. For 2D-NMR experiments, 45° pulse width was used within the standard CHEMPACK 4.1 pulse sequences (gDQCOSY, gHSQCAD, gHMBCAD). The heteronuclear single quantum coherence spectroscopy (HSQC) experiment was adjusted to ^1^*J*_CH_ = 146 Hz with DEPT-like editing and ^13^C-decoupling during acquisition time. In addition, the heteronuclear multiple bond correlation (HMBC) experiment was optimized for a long-range coupling of 8 Hz; a two-step ^1^*J*_CH_ filter was utilized (130–165 Hz).

### NMR data processing and quantification

The ^1^H NMR spectra were automatically fourier transformed, phase and baseline corrected with MestReNova software (9.0.1, Mestrelab Research, Santiago, Spain). The spectra were referenced to internal HMDS at 0.062 ppm for ^1^H NMR and to internal CD_3_OD signals at 49 ppm for ^13^C-NMR, respectively. Spectral intensities were then reduced to integrated sections of identical width (0.04 ppm), referred to as buckets, within the region of 10.94 to 0.0 ppm. The regions belonging to residual water between 5.0 and 4.7 ppm and methanol signals 3.26–3.20 ppm were removed prior and the data were subjected to multivariate analyses.

For metabolites quantification using ^1^H NMR spectroscopy (qNMR), the equation previously described by^19^ was followed. Manual integration of the peak areas of selected proton signals assigned to compounds and internal standard HMDS was performed.

### Headspace SPME volatiles analysis

Except for Long jack plus^®^, 20 mg of finely pulverized specimens were placed in an SPME screw cap vials and spiked with 2 µg (*Z*)-3-hexenyl acetate as an internal standard. The analysis of volatile compounds was carried out on a Schimadzu GC-17A gas chromatograph equipped with DB-5 column (30 m × 0.25 mm i.d. × 0.25 µm film thickness; Supelco) and coupled to Schimadzu QP5050A mass spectrometer. GC–MS analysis was performed following exact conditions previously reported in^20^. Two biological replicates for each group were analyzed under identical conditions for accounting the biological variability of the samples.

### GC–MS data processing

Volatile components were identified based on various paramenter, including their Kovat retention indices (KI) relative to C6-C20 n-alkane series, mass matching with the NIST and WILEY libraries, and with reference standards (when available). Before mass spectral matching, peaks were first deconvoluted through AMDIS software (http://www.amdis.net) and their peaks abundance data were extracted using MS-Dial tool (http://prime.psc.riken.jp/compms/msdial/main.html) applying the following parameters: retention time (0–20 min), mass range (0–1000 Da), accurate mass tolerance (MS^1^) 0.01, MS^2^, 0.025 Da, minimum peak height (1000), sigma (0.7), maximum charge number (2), MS^1^ tolerance for alignment (0.015 Da), and peak height 1000.

### Multivariate and univariate statistical analysis

The datasets generated from the NMR and GC–MS were subjected to multivariate data analysis. Prior to data analysis, normalization by pareto-scaling have been performed to allow adjustment of differences among the samples. Then, unsupervised exploratory analysis has been applied i.e. principal component analysis (PCA) and hierarchical clustering analysis (HCA) followed by supervised analysis i.e. orthogonal partial least squares-discriminant analysis (OPLS-DA) using SIMCA-P Version 13.0 (Umetrics, Umeå, Sweden). Differential variables were subsequently identified based on by analyzing the S-plot, which was declared with covariance (p) and correlation (pcor) in addition to the variable influence in the projection (VIP).

For the univariate analysis, the data are presented as mean ± SD. The quantified NMR metabolites were analyzed by one-way ANOVA followed by Dunnett's test for multiple group comparison of the commercial against the authentic samples (GraphPad Prism, version 8.0, GraphPad Software, La Jolla, CA). Differences were considered statistically significant when *p* ≤ 0.05. Volcano plots were employed for analysis of the the GC–MS metabolites under R version 4.1.0 where differential metabolites were subsequently identified based on compiling the results of unpaired two-tailed t-test with *p* values ≤ 0.05 and fold changes above 1.5 or below 0.66.

## Results and discussion

In the present study, a multiplexed approach of NMR and GC–MS metabolomics fingerprinting of *E.* *longifolia* root extract was employed to facilitate the identification and quantification of its major metabolites. Hence, reliable multivariate chemometric models can be developed for the authentication, standardization, and quality control of commercial *E.* *longifolia* preparations.

### NMR identification of *E. longifolia* metabolites

**Figure 1:**
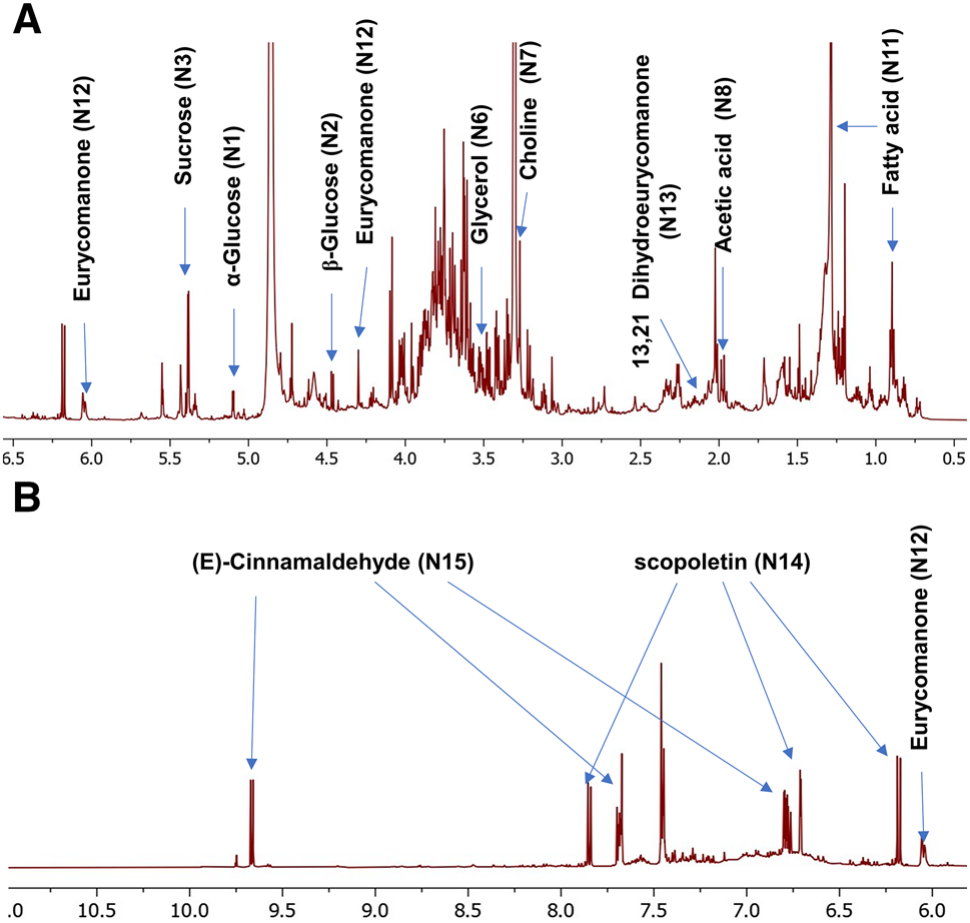
^1^H-NMR spectra of *E.* *longifolia* root extract **(A)** at *δ* 0.5–6.5 ppm, and **(B)** at *δ* 6.5–11.0 ppm showing characteristic signals for primary and secondary metabolites. Peaks assigned in the spectrum are labeled as listed in Table 1.

^1^H NMR analysis of *E.* *longifolia* root extract exhibited characteristic signals at two main regions; an up-field region (*δ*_H_ 0.5 to 6.0 ppm) of high intensity peaks ascribed mostly to sugars, organic and fatty acids in addition to quassinoids (Fig. 1A), and a down-field region (*δ*_H_ 6.0 to 9.5 ppm) with signals mainly belonging to aromatic compounds viz*.*, cinnamaldehyde and scopoletin (Fig. 1B). A total of 15 metabolites were identified (Fig. S1) mostly based on their 2D-NMR spectra to overcome overlapped peaks in such crude extract including ^1^H–^1^H COSY, ^1^H–^13^C HSQC and ^1^H–^13^C HMBC as listed in (Table 1).

**Table 1:** Resonance assignments with chemical shifts of constituents identified in 600 MHz ^1^H-NMR, HSQC and HMBC spectra of Tongkat Ali (*E.* *longifolia*) methanol extract. ^1^H-NMR quantification of most primary and secondary metabolites are expressed as μg/mg dry powder ± S.D (n = 3), see experimental section.

Metabolite	Assignment	*δ* ^1^H (ppm)	HSQC correlations *δ* ^13^C (ppm))	HMBC correlations *δ* ^13^C (ppm)
Sugars				
*α*-Glucose (N1)	C-1	5.10 (d, *J* = 3.6 Hz)	94.1	–
	C-2	3.36^a^	73.5	74.5(C3)
	C-5	3.68 (d, *J* = 2.4 Hz)	–	73.1(C4)
*β*-Glucose (N2)	C-1	4.47 (d, *J* = 7.87 Hz)	97.8	–
	C-2	3.13 (dd, *J* = 9.30, 7.87 Hz)	76.2	–
	C-5	3.31^a^	49.2	–
Sucrose (N3)	C-1	5.38 (d, *J* = 3.81 Hz)	93.5	74.5(C5), 105.1 (C2′)
	C-2	3.40^a^	73	74.5 (C3)
	C-3	3.707^a^	74.1	73.6(C2)
*α*-Fructofuranose (N4)	C-5	4.01 (d, *J* = 1.43 Hz)	73.1	71.1(C4)
*β*-Fructofuranose (N5)	C-3	4.10 (d, *J* = 8.34 Hz)	78.1	75.6 (C5)
	C-6	3.71^a^	63.7	72.9(C5)
Glycerol (N6)	C-1	3.58 (dd, *J* = 11.2, 4.8 Hz)	64.6	73.6 (C2)
	C-2	3.64^a^	73.8	–
Nitrogenous compounds				
Choline (N7)	*N*-(CH_3_)_3_	3.26^a^	54.7	53.7 (*N*-CH_3_), 67.3 (*N*-CH_2_)
	*N*-CH_2_	3.8^a^	67.3	53.7 (*N*-CH_3_)
Organic and fatty acids				
Acetic acid (N8)	C-2	1.97 (s)	21.9	176 (C1)
Succinic acid (N9)	C-2(α,β)	2.5^a^	32	–
Syringic acid (N10)	C-3/C-5	3.91^a^	56	149.3 (C3/5)
	C-2/C-6	7.03^a^	112	120.9(C1)
Fatty acid (N11)	C-1	–	175.1	
	C-2	2.33^a^	35.0	25.9(C3), 30.1(C4), 175.1(C1)
	C-3	1.60^a^	26.0	30.1(C3)
	Inner CH_2_	1.28–1.30 br. S	30–31	30.8 (inner CH_2_)
	ω-1	1.34^a^	23.5	30.2 (inner CH_2_)
	ω	0.9 (t, *J* = 7.15 Hz)	14.3	23.9(ω-1), 30.8(ω-2)
Quassinoids				
Eurycomanone (N12)	C-1	4.30 (s)	84.3	46.7 (C10), 198.8 (C2)
	C-3	6.05^a^	125.94	–
	C-5	3.041^a^	42.7	–
	C6	2.056^a^	26.2	130.8 (C4)
	C-7	4.72^a^	77.6	175.1 (C16)
	C-15	4.618^a^	71.8	175.1 (C16)
	C-21	5.43^a^	121.7	80.8 (C12), 79.34 (C20)
13,21 Dihydroeurycomanone (N13)	C-21	1.241^a^	10.8	42.5(C13), 84.3 (C12/C14)
	C-13	2.178^a^	–	–
Cinnamic and coumarin derivatives				
Scopoletin (N14)	C-2	6.18 (d, *J* = 9.4 Hz)	112.4	163.8(C1)
	C-3	7.85 (d, *J* = 9.4 Hz)	146.1	163.8(C1), 112.7(C2)
	C-8	6.71	103.3	113.8 (C4), 163.8(C1)
	OCH_3_	3.87	57.1	–
(*E*)-Cinnamaldehyde (N15)	C-1	9.66 (d, *J* = 7.87 Hz)	–	129.4(C2)
	C-2	6.78 (dd, *J* = 16.0, 7.87 Hz)	–	–
	C-3	7.68 (d, *J* = 15.5 Hz)	155	129.6 (C5/9)
	C5/C9	7.68^a^	130	132.6 (C 6/7/8)

^a^Overlapped signals.

Sugars were the dominant primary metabolites identified in *E.* *longifolia* root extract (Table 1) including α-glucose **(N1)**, *β*-glucose **(N2)**, sucrose **(N3)**, *α*-fructofuranose **(N4)**, *β*-fructofuranose **(N5)** and glycerol **(N6**). In detail, *α*- and *β*-glucose were assigned based on their anomeric protons’ signals displayed at *δ*_H_ 5.10 (d, *J* = 3.6 Hz) and 4.47 (d, *J* = 7.9 Hz), respectively. These signals showed cross peaks correlations with their corresponding carbons at *δ*^13^C of 94.1 and 97.8 ppm in the ^1^H-^13^C HSQC spectra. Sucrose was identified based on its characteristic anomeric proton at *δ*_H_ 5.38 (d, *J* = 3.81 Hz) and further confirmed by examining anomeric proton 2D-NMR correlations, i.e., COSY with cross peak at *δ*_H_ 3.40 and TOCSY with cross peak at *δ*_H_ 3.71 for H-2 and H-3 of the glucose moiety, respectively. In addition, α-fructofuranose and *β*-fructofuranose were identified from their H-5 and H-3 signals resonating as a doublet at *δ*_H_ 4.01 (*J* = 1.4 Hz) and at *δ*_H_ 4.10 (*J* = 8.34 Hz), respectively. Sugar alcohol exemplified by glycerol was identified based on its signal at *δ*_H_ 3.58 assigned to the diastereotropic methylene protons at C-1 position of glycerol (Fig. S1) and to contribute to roots sweet taste.

NMR analysis also revealed the presence of many organic acids including acetic acid (**N8**), succinic acid (**N9**), and syringic acid (**N10**) in agreement with reported literature^21^. Acetic acid and succinic acid were identified based on their corresponding singlet protons at *δ*_H_ 1.97 and 2.50 ppm with HSQC cross-peak correlation at *δ*^13^C 21.9 and 32 ppm, respectively. Another phenolic acid identified as syringic acid displayed characteristic signal for its methoxy protons at *δ*_H_ 3.91 showing correlation to *δ* ^13^C 56 in the HSQC spectra and long-range coupling to the aromatic methine carbons at *δ* ^13^C 149.3 in HMBC spectra. Other than organic acids, few peaks for fatty acids (**N11**) could be readily assigned in the ^1^H NMR spectrum such as two signals at *δ*_H_ 0.90 and *δ*_H_ 1.28–1.30 ppm for the terminal methyl and repeated methylene of fatty acids, respectively.

Compared to primary metabolites to impart tongkat ali typical flavor, quassinoids, the major secondary metabolites of *E.* *longifolia* roots were detected in this study including eurycomanone (**N12**) and 13,21-dihydroeurycomanone (**N13**) and to account more for roots hormonal effects in agreement with reported literature^21^. Eurycomanone was readily assigned from characteristic signal of its olefinic proton H-3 at *δ*_H_ 6.05 which exert cross peak correlation to its carbon at *δ* ^13^C 125.9 ppm in the HSQC spectra (Fig. S1). In addition, the deshielded signal of H-7 at *δ*_H_ 4.72, showed a long-range coupling with the carbonyl carbon (C-16) at *δ* ^13^C 175.1 ppm in HMBC and cross peak correlation to its direct carbon at *δ* ^13^C 77.6 ppm in HSQC spectra. Interestingly, H-21 signal of eurycomanone can be identified as the differential signal from that of 13,21-dihydroeurycomanone (Fig. S1). This signal was observed at *δ*_H_ 5.43 due to the presence of olefinic double bond (C13 = C21) for eurycomanone, while in 13,21-dihydroeurycomanone signal appeared at *δ*_H_ 1.241. It has been reported that both compounds displayed significant increase in spermatogenesis mostly via direct effect on the hypothalamic–pituitary–gonadal axis which aid in the fertility-enhancing property of the *E.* *longifolia*^22^.

Compared to quassinoids as major secondary metabolites in *E.* *longifolia*, aromatic region showed key signals in the downfield region 6.5–10.0 ppm for cinnamates ascribed to scopoltin (**N14**) and (*E*)-cinnamaldehyde (**N15**), respectively and to add to roots unique flavor as key components. Scopoltin, a hydroxycoumarin with 6-methoxy substitution previously identified in *E.* *longifolia*^23^, was identified from its characteristic olefinic protons H-2 and H-3 (Fig. S1) appearing as a pair of doublets at *δ*_H_ 6.18 and 7.85 ppm (*J* = 9.4), respectively. These peaks displayed distinct correlation signals in ^1^H-^1^H COSY experiment, and cross peak correlations with the carbonyl carbon (C-1) at *δ* ^13^C 163.8 ppm in the HMBC spectra confirming the coumarin nucleus. The methoxy group substitution was detected at *δ*_H_ 3.87 ppm correlated to *δ* ^13^C 57.1 ppm in HSQC spectra. Cinnamaldehyde, a major aroma compound in cinnamon, was detected for the first time in *E.* *longifolia* based on its three-spin systems *i.e.* a doublet aldehyde peak at *δ*_H_ 9.66 (*J* = 7.87 Hz), olefinic signals at *δ*_H_ 6.78 (dd, *J* = 16.0, 7.87 Hz) and 7.68 (d, *J* = 15.5) corresponding to olefinic H-2 and H-3, respectively (Fig. S1)^24^. The remaining aromatic protons were observed as multiplets at *δ*_H_ 7.45/7.68 ppm and further confirmed using HSQC and HMBC experiments (Table 1). The 16.0 Hz coupling of the olefinic protons indicated its *(E)*-geometry.

### Multivariate data analysis of *E. longifolia* commercial preparations based on ^1^H NMR dataset

**Figure 2:**
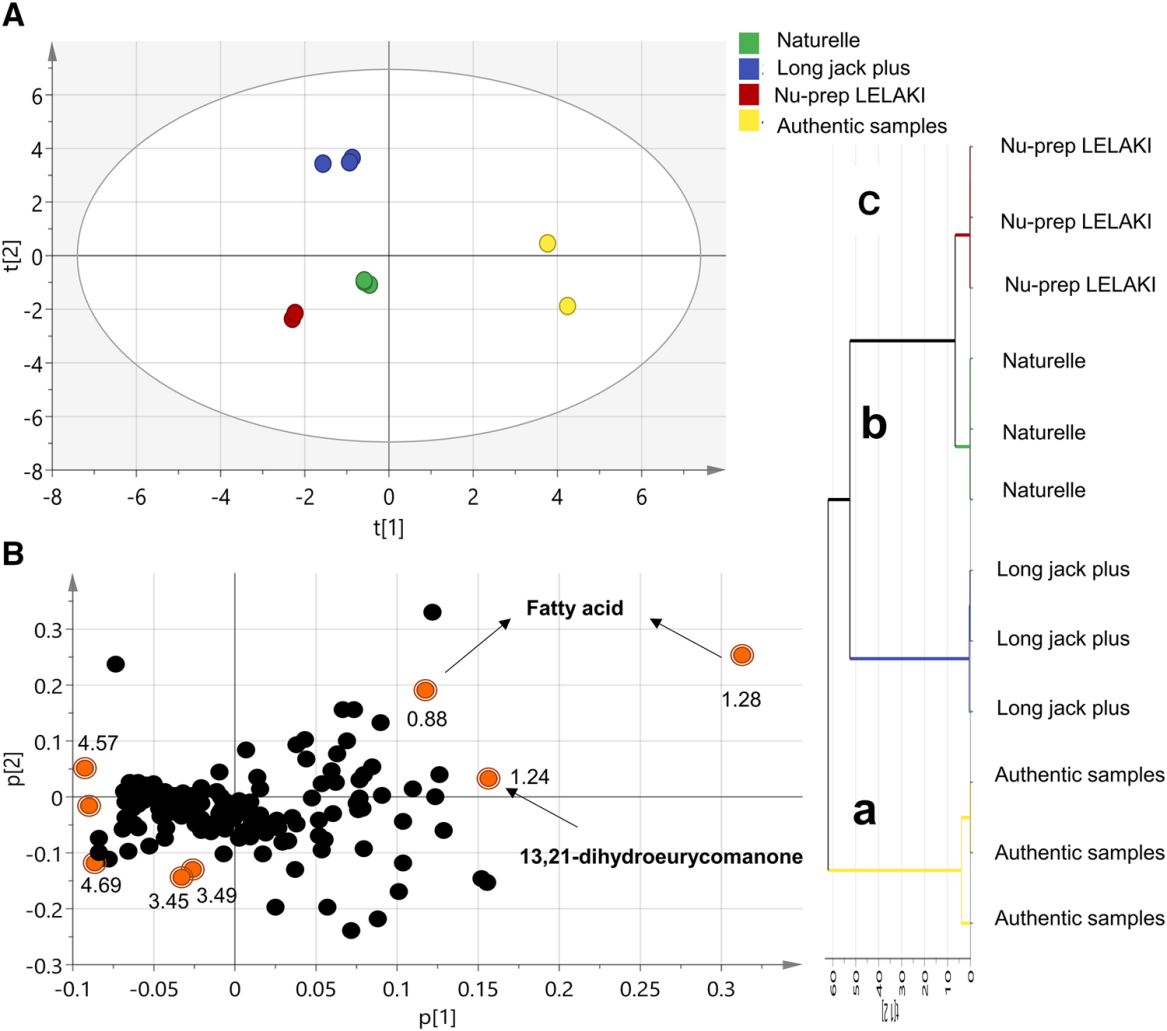
Principal component analysis (PCA) and hierarchical cluster analysis (HCA) of *E.* *longifolia* commercial preparations NMR dataset. (**A)** Score plot of PC1 vs*.* PC2, complete segregation of the authentic samples from the commercial products. (**B)** Loading plot for PC1 and PC2 showing signals of fatty acids (*δ* 0.88, 1.28 ppm) and 13,21-dihydroeurycomanone (*δ* 1.24 ppm) as the major signals contributing to samples discrimination. (**C**) HCA of NMR dataset shows two main cluster: cluster (a) for the authentic samples and cluster (b) for the commercial product specimens.

To further determine how commercial preparation NMR fingerprint compare to authenticated tongkat ali root, three of its commercial preparations i.e., Naturelle^®^, Long jack plus^®^, and Nu-prep LELAKI^®^ were modelled alongside roots based on their acquired NMR dataset. Details on products´ composition are presented in the experimental section. PCA was first employed to determine the pattern among examined samples in an unsupervised manner. The score plot revealed for complete segregation of authenticated root from commercial products suggesting for metabolic differences with PC1 and PC2 accounting for 89.23% of the variance (Fig. 2A). NMR signals that had major contribution to such pattern can be identified from the corresponding loading plot with peaks of fatty acids (*δ*_H_ 0.88, 1.28 ppm) and 13,21-dihydroeurycomanone (*δ*_H_ 1.24 ppm) found more enriched in authentic root as evident from their positive PC1 values (Fig. 2B). The signals related to commercial products (*δ*_H_ 3.45–4.69 ppm) appeared to belong to sugars in the upper-mid ^1^H NMR spectra. Similar results were also observed from HCA dendrogram (Fig. 2C) with two main cluster: cluster (a) for authentic root and cluster (b) for commercial product samples.

**Figure 3:**
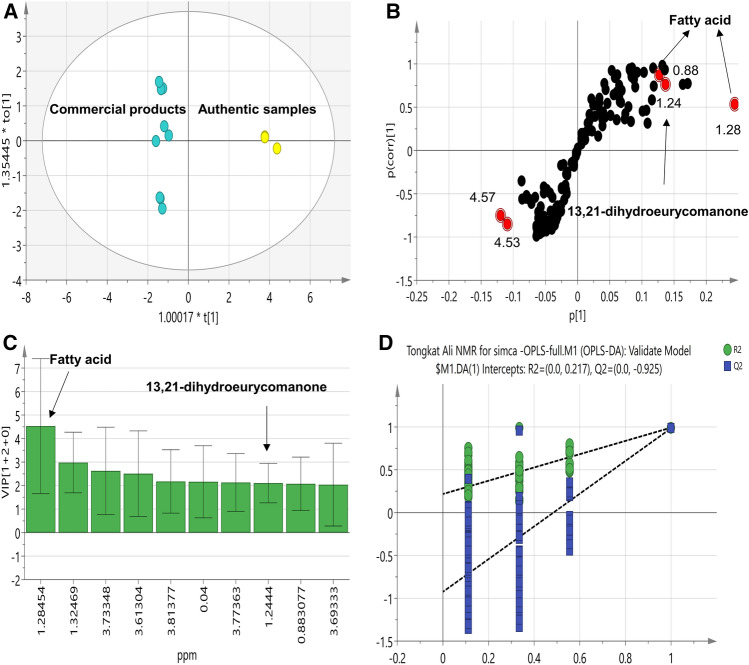
**(A)** OPLS-DA score plot and (**B)** loading S-plots derived from *E.* *longifolia* authentic root modelled against other commercial samples analysed by ^1^H-NMR, n = 3**.** The p(corr)[1] axis represents the correlation of the bin towards the predictive variation. The p[1] axis represents the magnitude of the spectral bins. (**C)** Top 10 variables that have the greatest influence on the projection as revealed from the developed OPLS-DA. (**D)** Permutation test (n = 200) as a validation criterion for the OPLS-DA model, the test verifies the validity of the model with negative intercept for the Q2 values.

Another multivariate analysis model i.e. OPLS-DA was employed for confirming PCA results and identifying markers. OPLS-DA exhibit its advantage from finding the maximum separation among the studied classes and identifying differential biomarkers in a supervised approach. In agreement with PCA results, complete separation was attained along the predictive components between the authentic samples and the commercial products with a fitness of data R2(X) of 94.8%, R2(Y) of 99.2% and predictive ability Q2 of 98.5% (Fig. 3A). Metabolites that influence such pattern were verified form loading S-plot (Fig. 3B) and VIP plot (Fig. 3C) where peaks related to fatty acids (*δ*_H_ 0.88, 1.28 ppm) and 13,21-dihydroeurycomanone (*δ*_H_ 1.24 ppm) were found abundant in authenticated root compared to the commercial products in agreement with the PCA results. Regarding validity of the developed model, a valid model was obtained using permutation test with negative Q2 intercept value (Fig. 3D) and CV-ANOVA with *p* < 0.05.

### ^1^H NMR-based quantification of identified metabolites

The absolute concentrations of identified metabolites were determined both in the authentic root and commercial products to aid in standardization of *E.* *longifolia* nutraceuticals. Out of the 15 identified metabolites, 10 metabolites (7 primary and 3 secondary metabolites) were successfully quantified considering they showed no signal overlap, where quantification and precise metabolites measurement were calculated as μg/mg extract based on the integration of their corresponding peaks in the ^1^H NMR spectra as shown in (Table S1). Representative ^1^H NMR spectra of the commercial preparations i.e., Naturelle^®^, Long jack plus^®^, and Nu-prep LELAKI^®^ is depicted in (Fig. S2).

The authentic *E.* *longifolia* extract showed α- and β-glucose at 2.8 µg/mg and 4.3 µg/mg, respectively. However, commercial products, especially Nu-prep LELAKI^®^ showed the highest contents at 38.0 and 49.0 µg/mg, while the Long jack plus^®^ contained the lowest levels at 1.1 and 3.8 µg/mg, respectively. β-Glucose is the building monomer of cellulose which is the major polysaccharide in plant cell walls. Interestingly, the β-glucose showed non-significant difference with the authentic root at *p* < 0.05. Also, a similar pattern was observed with other primary metabolites such as sucrose, α-fructofuranose, β-fructofuranose, glycerol, and acetic acid. Moreover, the highest and significant content of β-fructofuranose was detected in Naturelle^®^ and Nu-prep LELAKI^®^ products at 28.3 and 15.1 µg/mg, respectively, at more than fivefold levels compared to authentic roots suggestive that industrial processing to lead to more sweet taste in products. In contrast, the sweet taste of authentic roots may originate from the highest sucrose content (14.0 µg/mg).

On the other hand, the secondary metabolites scopoletin and (*E*)-cinnamaldehyde demonstrated the highest levels in authenticated root at 4.7 and 1.9 µg/mg, respectively, with a significant difference (*p* < 0.05) compared to other commercial products. However, the other identified quassinoid eurycomanone was detected at higher levels in commercial products, specifically in Naturelle^®^ and Nu-prep LELAKI^®^ at 37.0 and 18.9 µg/mg, respectively. The richness of commercial products in eurycomanone is recognized as a superior objective and encourages better marketing of the tongkat ali herbal products as sexual function and fertility-promoting products helping to improve serum testosterone levels^25^. Furthermore, the lowest abundance of quassinoids in Long jack plus^®^ preparations proposed its nutritional and medicinal effects resulting from the combined herbal mixture present in its constituents.

Since the root is the organ of storage of metabolites that provide energy source. The investigated samples showed richness in sugars and their products of metabolism. These findings might be inferred from the low abundance either in primary sugars or secondary metabolites in Long jack plus^®^ product which contains additional plant extracts other than tongkat ali root, including *Nigella* *sativa* seeds. Nevertheless, other differences of authentic roots compared with Naturelle^®^ and Nu-prep LELAKI^®^ are likely attributed to the processing protocols and excipients added during manufacturing. Yet, other products from different companies worldwide and authentic samples from different geographical origins ought to be analyzed to provide insights into the further processing procedures followed by companies.

### GC–MS based aroma profiling of *E. longifolia* and its commercial preparations

Considering that cinnamaldehyde appeared as major component in *E.* *longifolia* using NMR, aroma profiling was attempted using more sensitive technique targeting volatile organic compounds (VOCs) using SPME coupled with GC–MS in roots as a more sensitive technique alongside two of its available commercial products, i.e., Nu-prep LELAKI^®^ and Naturelle^®^. However, Long jack plus^®^ specimen was excluded from aroma profiling by SPME coupled with GC–MS since such product contained other aromatic herbal extracts, including *N.* *sativa* and *Cassia* *angustifolia*, which might interfere with tongkat ali original aroma constituents giving unreliable results.

This study assessed tongkat ali aroma directly from the authentic roots powder in contrast to the previous studies and likewise the effect of industrial processing and pharmaceutical formulation, including excipients, which have not been investigated yet. Previous aroma analysis revealed a number of VOCs extracted by either water or methanol and detected by electronic nose response and SPME coupled with GC–MS. However, non-specific volatiles as 2-hexadecanol, nonanal, acetaldehyde, acetic acid, 2-methylhexanol and 2(5*H*)-furanone were identified^6,26,27^.

**Figure 4:**
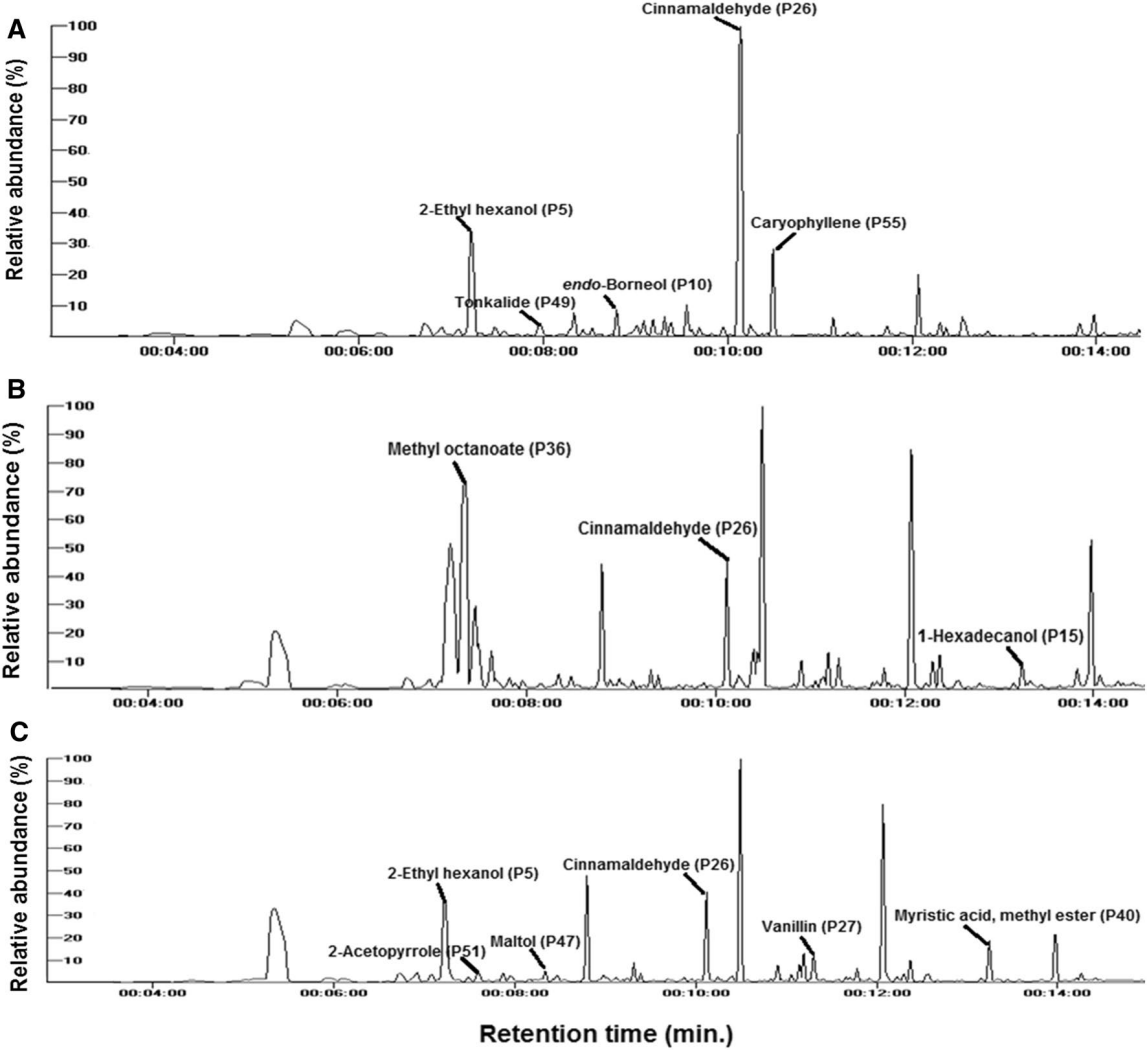
GC–MS total ion chromatograms (TIC) of investigated *Eurycoma* *longifolia* (Tongkat Ali) products showing the relative abundances (%) of each identified peaks of; (**A)** authentic root powder, (**B)** Nu-prep LELAKI^®^, and (**C)** and Naturelle^®^ specimens highlighting major aroma component in each specimen.

The results of the current study showed that the GC total ion chromatograms (TIC) of the three investigated samples, i.e., *E.* *longifolia* and Nu-prep LELAKI^®^, and Naturelle^®^ products showed relatively different patterns (Fig. 4). A total of 59 VOCs were identified listed in Table 2 belonging to acids (3 compounds), alcohols (14), aldehyde/furans (10), aliphatic hydrocarbons (3), aromatic hydrocarbons (1), phenol/ethers (4), fatty acids/esters (9), ketones (4), lactones (2), nitrogenous compounds (2), and sesquiterpene hydrocarbons (7). A total of 133 volatiles were previously identified in *E.* *longifolia* extracts via SPME (carboxen/polydimethylsiloxane fiber) using a smell sensor equipped with a gas chromatographic stationary phases and lipid materials similar to the olfactory system^27^. Difference in volatiles recovery may be attributed to such pre-treatment for volatiles collection and the higher number and diversity of analyzed samples, i.e., 10 samples of freeze-dried and spray-dried. Fatty acids/esters and aldehydes/furans amounted for the most abundant classes in investigated samples and in agreement with NMR results revealing for predominance of aldehydes i.e., *E*-cinnamaldehyde. Authentic powder and Naturelle^®^ were rich in aldehydes/furans at 77.1% and 54.8% of total aroma compounds, respectively. In contrast, Nu-prep LELAKI^®^ was found to be enriched in fatty acids/esters (74.6%), Table 2. These findings indicated that industrial processing and/or pharmaceutical formulation affect VOCs profile to various extent in investigated products of tongkat ali. The following subsections shall highlight the major classes of volatiles and differences observed among samples.

**Table 2:** Relative abundances (%) ± SD of volatiles detected in tongkat ali dried powder (*Eurycoma* *longifolia*) analyzed via SPME coupled with GC/MS in comparison with the marketed products, i.e., Nu-prep LELAKI and Naturelle (n = 2).

Peak no.	Name	Rt (min)	RI	Chemical class	Relative abundances (%)		
					Tongkat ali (*E.* *longifolia*)	Nu-prep LELAKI (Del Aki^®^)	Naturelle®
1.	Hexanoic acid	6.919	977	Acids	0.05 ± 0.0	0.01 ± 0.0	0.13 ± 0.1
2.	Heptanoic acid	7.939	1076		0.00 ± 0.0	0.02 ± 0.1	0.03 ± 0.0
3.	Heptanoic acid	9.907	1266		0.00 ± 0.0	0.23 ± 0.0	0.01 ± 0.0
Total acids					0.05 ± 0.0	0.26 ± 0.1	0.16 ± 0.0
4.	(*Z*)-3-Hexen-1-ol	5.285	784	Alcohols	0.08 ± 0.1	0.19 ± 0.1	0.40 ± 0.0
5.	2-Ethyl hexanol	7.469	1033		2.83 ± 0.1	1.43 ± 1.0	2.58 ± 0.0
6.	Benzyl alcohol	7.573	1042		0.07 ± 0.0	0.04 ± 0.0	0.18 ± 0.0
7.	1-Octanol	7.932	1076		0.79 ± 0.6	0.42 ± 0.2	1.34 ± 0.7
8.	α-Terpineol	7.954	1077		0.03 ± 0.0	0.14 ± 0.1	0.51 ± 0.0
9.	Cinnamyl alcohol	9.061	1180		1.11 ± 0.2	0.09 ± 0.0	0.51 ± 0.0
10.	*endo*-Borneol	9.071	1181		3.27 ± 0.5	0.22 ± 0.0	0.75 ± 0.2
11.	Terpinen-4-ol	9.177	1190		1.80 ± 0.1	0.15 ± 0.0	0.40 ± 0.1
12.	3-Phenylpropanol	9.675	1242		1.39 ± 0.3	0.15 ± 0.2	0.68 ± 0.3
13.	1-Dodecanol	11.861	1468		0.32 ± 0.0	0.09 ± 0.0	0.22 ± 0.0
14.	α-Cadinol	12.609	1542		0.09 ± 0.1	0.02 ± 0.0	0.18 ± 0.1
15.	1-Hexadecanol	13.132	1593		0.30 ± 0.0	1.05 ± 0.2	0.20 ± 0.0
16.	Gleenol	13.281	1607		0.21 ± 0.0	0.38 ± 0.1	0.15 ± 0.0
17.	Cubenol	13.81	1666		0.30 ± 0.0	1.05 ± 0.2	0.20 ± 0.0
Total alcohols					12.58 ± 2.3	5.43 ± 2.2	8.31 ± 1.6
18.	Benzaldehyde	6.737	956	Aldehydes/furans	1.22 ± 0.3	0.25 ± 0.0	0.45 ± 0.0
19.	Nonanal	6.977	983		0.50 ± 0.2	0.18 ± 0.0	0.50 ± 0.2
20.	*trans*-2-Nonenal	7.568	1043		0.67 ± 0.1	0.28 ± 0.0	0.99 ± 0.2
21.	Benzene propanal	7.638	1049		0.03 ± 0.0	0.02 ± 0.0	0.06 ± 0.0
22.	Benzene acetaldehyde	7.689	1054		0.76 ± 0.1	1.14 ± 0.1	1.51 ± 0.1
23.	Alletone/furaneol	7.786	1062		0.00 ± 0.0	1.03 ± 0.0	0.46 ± 0.0
24.	Undecanal	9.014	1176		0.49 ± 0.1	0.05 ± 0.0	0.47 ± 0.0
25.	Lauraldehyde	9.11	1184		0.11 ± 0.0	0.32 ± 0.1	0.45 ± 0.1
26.	Cinnamaldehyde	10.113	1288		72.97 ± 3.8	8.08 ± 5.8	39.13 ± 0.1
27.	Vanillin	11.289	1412		0.35 ± 0.2	2.94 ± 0.3	10.80 ± 2.5
Total aldehyde/furans					77.09 ± 4.8	14.29 ± 6.4	54.83 ± 3.2
28.	3-Methyl pentadecane	12.872	1567	Aliphatic hydrocarbons	0.01 ± 0.0	0.01 ± 0.1	0.09 ± 0.1
29.	*n*-Hexadecane	13.229	1602		0.01 ± 0.0	0.00 ± 0.0	0.01 ± 0.0
30.	*n*-Heptadecane	14.245	1713		0.12 ± 0.0	0.22 ± 0.5	1.17 ± 0.5
Total aliphatic hydrocarbons					0.13 ± 0.0	0.23 ± 0.1	1.27 ± 0.5
31.	2-Methyl naphthalene	9.218	1194	Aromatic hydrovarbons	0.0 ± 0.0	0.01 ± 0.0	0.05 ± 0.0
Total aromatics					0.0 ± 0.0	0.01 ± 0.0	0.05 ± 0.0
32.	Guaiacol	8.204	1101	Phenols/ethers	0.05 ± 0.0	0.08 ± 0.0	0.37 ± 0.0
33.	Safrole	8.943	1169		0.00 ± 0.0	0.02 ± 0.0	0.06 ± 0.1
34.	4-Acetylanisole	10.864	1369		0.34 ± 0.2	0.32 ± 0.1	0.81 ± 0.2
35.	Methyleugenol	11.253	1408		0.04 ± 0.0	0.12 ± 0.0	0.27 ± 0.1
Total phenol/ethers					0.43 ± 0.2	0.55 ± 0.1	1.51 ± 0.4
36.	Methyl octanoate	7.168	1006	Fatty acids/esters	1.28 ± 0.2	74.02 ± 7.2	2.47 ± 0.3
37.	Methyl *n*-nonanoate	8.19	1098		0.13 ± 0.0	0.16 ± 0.0	0.93 ± 0.1
38.	Lauric acid, methyl ester	11.035	1385		0.04 ± 0.0	0.01 ± 0.0	0.04 ± 0.0
39.	Cinnamyl acetate	11.648	1447		0.10 ± 0.0	0.22 ± 0.3	0.91 ± 0.3
40.	Myristic acid, methyl ester	13.121	1591		0.00 ± 0.0	0.00 ± 0.0	8.20 ± 5.8
41.	Methyl palmitoleate	14.636	1756		0.02 ± 0.1	0.03 ± 0.0	0.09 ± 0.0
42.	Myristic acid	14.718	1765		0.11 ± 0.2	0.06 ± 0.0	0.56 ± 0.3
43.	Palmitic acid, methyl ester	14.835	1779		0.03 ± 0.0	0.01 ± 0.0	0.05 ± 0.0
44.	Pentadecanoic acid	15.544	1854		0.05 ± 0.1	0.04 ± 0.0	0.34 ± 0.2
Total fatty acids/esters					1.75 ± 0.4	74.56 ± 7.5	13.59 ± 7.1
45.	2(5*H*)-Furanone	6.094	879	Ketones	0.00 ± 0.0	0.01 ± 0.0	0.01 ± 0.0
46.	Furfuryl hydroxymethyl ketone	8.13	1094		0.02 ± 0.0	0.06 ± 0.0	0.08 ± 0.0
47.	Maltol	8.451	1124		0.02 ± 0.0	1.67 ± 0.2	4.64 ± 0.4
48.	Benzophenone	12.465	1527		0.04 ± 0.0	0.02 ± 0.0	0.07 ± 0.0
Total ketones					0.08 ± 0.0	1.76 ± 0.2	4.81 ± 0.4
49.	Tonkalide	7.797	1064	Lactones	0.39 ± 0.1	0.59 ± 0.1	1.51 ± 0.2
50.	γ-Amyl-butyrolactone	10.931	1375		0.45 ± 0.1	0.13 ± 0.0	1.20 ± 0.3
Total lactones					0.84 ± 0.1	0.7 ± 0.1	2.7 ± 0.4
51.	2-Acetylpyrrole	7.86	1069	Nitrogenous compounds	0.27 ± 0.1	0.91 ± 0.1	6.22 ± 1.1
52.	Benzyl isothiocyanate	11.004	1383		0.92 ± 0.9	0.23 ± 0.2	1.86 ± 1.0
Total nitrogenous compounds					1.19 ± 1.0	1.13 ± 0.4	8.08 ± 2.1
53.	α-Copaene	9.824	1257	Sesquiterpene hydrocarbons	0.08 ± 0.0	0.19 ± 0.1	0.55 ± 0.2
54.	Sativene	10.009	1277		0.17 ± 0.0	0.04 ± 0.0	0.36 ± 0.1
55.	β-Caryophyllene	10.228	1301		2.99 ± 2.7	0.26 ± 0.2	2.46 ± 1.3
56.	4,5,9,10-Dehydro-isolongifolene	11.387	1422		0.03 ± 0.0	0.02 ± 0.0	0.08 ± 0.0
57.	*α*-Muurolene	12.288	1509		0.07 ± 0.0	0.26 ± 0.1	0.05 ± 0.0
58.	Cadinene	12.525	1533		1.65 ± 0.3	0.15 ± 0.1	0.74 ± 0.2
59.	Calacorene	12.803	1560		0.88 ± 0.1	0.15 ± 0.0	0.45 ± 0.1
Total sesquiterpene hydrocarbons					5.87 ± 3.2	1.06 ± 0.4	4.68 ± 1.9

### Aldehydes/furans

Aldehyde/furan compounds were represented by ten metabolites ranging from 14.3% in Nu-prep LELAKI product to 77.1% in authentic powder. However, in the Naturelle product they amounted to 54.8% (Table 2). The high abundance of aldehyde/furan in the authentic powder was attributed to *E*-cinnamaldehyde (P26) which accounted for 73.0% of the total volatiles’ abundance, Fig. 4A, and in agreement with NMR results, Fig. 1B and Table 1. In contrast, (*E*)-cinnamaldehyde was detected at much lower levels in commercial preparations 8.1% and 39.1% in Nu-prep LELAKI and Naturelle commercial products, Fig. 4B,C. Decrease was also observed in other aldehydes including benzaldehyde (P18) detected at 1.22% in authentic root powder versus 0.45% and 0.25% in Naturelle^®^ and Nu-prep LELAKI, respectively. In contrast, alletone/furaneol (P23) which is a furan derivative detected in commercial products and was completely absent in authentic powder, Table 2. The presence of this compound may indicate its production during the processing step from heating of sugars.

Aldehydes showing opposite accumulation pattern included vanillin detected at 10.8% and 3.0% in Naturelle^®^ and Nu-prep LELAKI^®^ products, respectively versus only 0.35% in authenticated root powder. The last finding may be linked with the addition of vanillin-containing flavor for taste improvement. The results were in agreement with previous reports for aroma extraction using soxhlet extraction of tongkat ali root, where vanillin (2.22%) and benzaldehyde (0.87%) were among the identified aldehydes^6^.

### Alcohols

Alcohols were represented by 14 compounds, which were the most diverse chemical class ranging from 5.4% in Nu-prep LELAKI to 12.6% in authentic *E.* *longifolia* powder represented by *endo*-borneol (P10), 2-ethyl hexanol (P5), and terpinen-4-ol (P11) at 3.3%, 2.8%, and 1.8%, respectively (Table 2). *endo*-Borneol or isoborneol and terpinen-4-ol are monoterpene alcohols to possess a fragrant odor and^28,29^. The identification of both compounds in tongkat ali is reported for the first time and likely to contribute to the overall aroma in *E.* *longifolia* root.

However, in other commercial preparations, i.e., Nu-prep LELAKI^®^ and Naturelle^®^, *endo*-borneol (P10) was not a major alcohol component, while 2-ethyl hexanol (P5) was the most abundant alcohol detected at 1.4% and 2.6%, respectively (Table 2).

Previous reports showed only 1-propanol and 1-hexadecanol as volatile alcohols in tongkat ali extracted by conventional steam extraction^6^, in addition to 2-hexadecanol and 2-methylhexanol extracted by microwave-assisted extraction^26^. These findings infer that SPME coupled with GC–MS is more efficient for volatiles extraction and providing the true aroma of medicinal herbs.

### Fatty acids/esters

Fatty acids/esters were also detected as major constituents especially in commercial products amounting for 74.6 and 13.6% for Nu-prep LELAKI^®^ and Naturelle^®^, respectively and less abundant in root powder (Table 2). 9 Compounds were identified including methyl octanoate (P36), Fig. 4B as major form detected at 74.0% in Nu-prep LELAKI especially compared to other specimens, Table 2. Methyl octanoate is a major food flavoring agents possessing a winey, fruity, orange odour^30^ and is likely to be added in the commercial product of Nu-prep LELAKI^®^ intensifying the product's odour though not indicated on its label. Other esters, including 2-propyl tetradecyl ester and 1-methylundecyl ester previously reported^26^ were found absent in this study. These findings could be attributed to different extraction methods. Moreover, myristic acid methyl ester (P40) was found to be rich in Naturelle^®^ preparation at 8.2% and absent in authentic and Nu-prep LELAKI^®^ products.

These differences pointed out that industrial processing and formulation affected volatile profile, especially fatty acid/ester class in tongkat ali preparation. Furthermore, radar plot (Fig. S3) showed the unique aroma profile of Nu-prep LELAKI^®^ and its richness in fatty acid/ester and different from authentic root powder and Naturelle^®^ which were abundant in aldehyde/furan, i.e., *E*-cinnamaldehyde. SPME results fall in accordance with NMR results, Table 2.

### Sesquiterpene hydrocarbons

Sesquiterpene hydrocarbons (C_15_) represented another major class, Table 2, detected at comparable levels ca. 5% in authentic root and Naturelle^®^. β-Caryophyllene (P55) was the most abundant within that class amounting for 3.0%, 2.5%, and 0.3% in the authentic root, Naturelle^®^, and Nu-prep LELAKI^®^, respectively.

### Miscellaneous

Other classes were also detected and may contribute to root aroma and flavor. Particularly, the ketone compound maltol (P47) at 4.6% and nitrogenous compound 2-acetylpyrrole (P51) at 6.2% were more abundant in Naturelle^®^ preparation in contrast to other investigated samples (Table 2). Maltol was also found in the other commercial product Nu-prep LELAKI^®^ though at lower levels 1.7%. Maltol exhibits a fragrant caramel-butterscotch odor, while 2-acetylpyrrole is a Maillard reaction product, which is associated with foods drying/roasting and contributes to herbal aroma^31^. The presence of these compounds can be used as an index for thermal processing and drying step level in tongkat ali. Finally, tonkalide (P49) was detected in all samples at relatively low levels ranging from 0.4 to 1.5%. To the best of our knowledge, these volatiles are identified in tongkat ali for the first time either in authentic or commercial products.

In conclusion, the aroma of the authentic tongkat ali root is mainly attributed to cinnamaldehyde, *endo*-borneol, and β-caryophyllene, while Naturelle^®^ preparation is a mixture of volatile constituents acquired during industrial formulation from cinnamaldehyde, vanillin, maltol, and 2-acetylpyrrole. Whereas Nu-prep LELAKI^®^ preparation showed the least similar aroma profile to that of tongkat ali being dominated by methyl octanoate. These findings are in accordance with that of NMR quantification, which showed the less quality of Nu-prep LELAKI^®^ compared with Naturelle^®^, Table S2. Also, analysis of commercial preparations from other origins shall aid identifying how formulation and/or processing steps can impact root aroma attributes.

### Multivariate data analysis of tongkat ali commercial preparations based on GC–MS dataset

**Figure 5:**
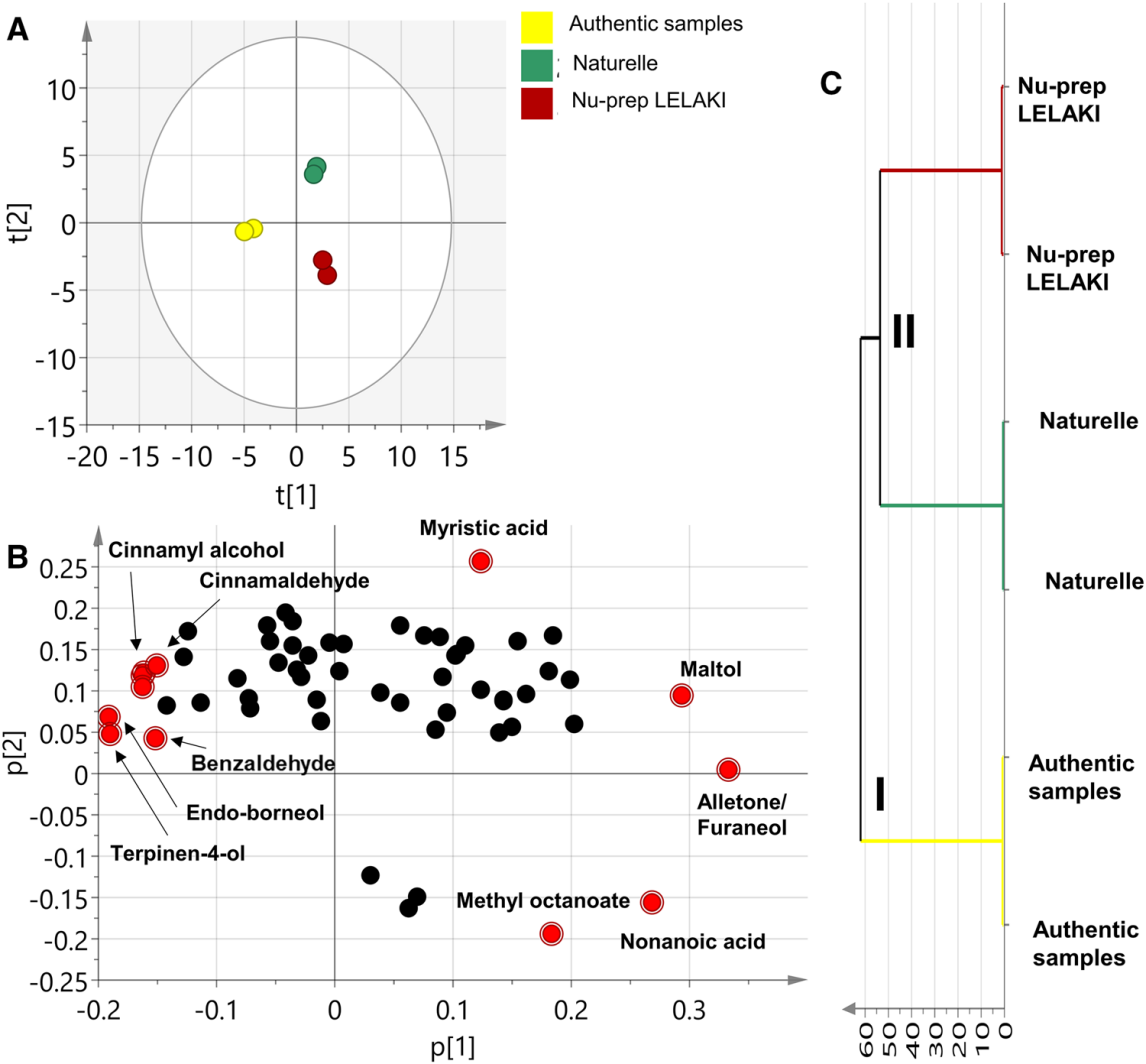
Principal component analysis (PCA) and Hierarchical cluster analysis (HCA) of *E.* *longifolia* commercial preparations GC–MS dataset. (**A)** Score Plot of PC1 vs. PC2. (**B)** Loading plot for PC1 and PC2 showing features the have the major contributing to samples discrimination. (**C)** HCA of GC–MS dataset shows two main cluster: cluster (I) for the authentic samples and cluster II) for the Nu-prep LELAKI^®^ and Naturelle^®^ samples signifying their heterogeneity and compositional differences.

Multivariate data analysis of the GC–MS dataset was also employed to assess compositional differences between tongkat ali authentic roots and its two commercial preparations i.e., Naturelle^®^ and Nu-prep LELAKI^®^ based on their VOC profiles. A similar segregation pattern to NMR model (Fig. 2) was observed confirming the validity of the findings. PCA score plot showed that authentic samples were completely segregated away from commercial products along the first principal component with negative PC1 values (Fig. 5A). In contrast, commercial products with positive PC1 values were separated along the second components (PC2) from each other with both PC1 and PC2 accounting for 80.3% of the total variance (Fig. 5A). In terms of VOCs that signify such pattern, loading plot revealed that signals related to benzaldehyde, *endo*-borneol and terpinen-4-ol in addition to cinnamates were enriched in the authentic root (Fig. 5B) in agreement with results presented in (Table 2). Asides, peaks related to methyl octanoate and nonanoic were more enriched in the Nu-prep LELAKI^®^ preparation while peaks related to maltol and myristic acid were more abundant in Naturelle^®^ samples and posing these volatiles as potential differential biomarkers for tongkat ali QC analysis in the future. HCA analysis comes in agreement with PCA results with two main clusters: cluster (I) for the authentic root and cluster (II) for the Nu-prep LELAKI^®^ and Naturelle^®^ samples signifying their heterogeneity and compositional differences in terms of their VOCs with the authentic group (Fig. 5C).

**Figure 6:**
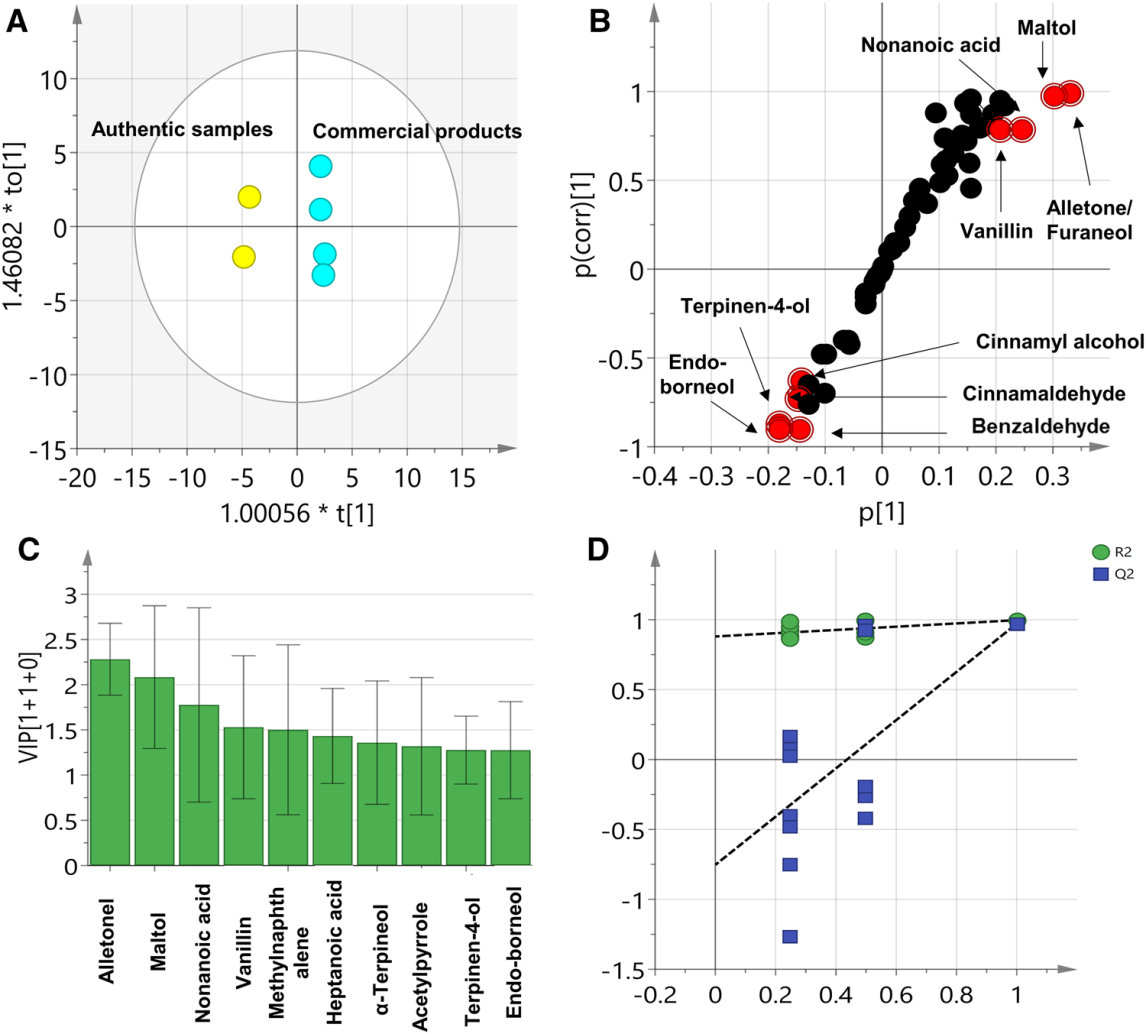
**(A)** OPLS-DA score plot and (**B)** loading S-plots derived from *E.* *longifolia* authentic samples modelled against other commercial samples analysed by GC–MS**. (C)** top 10 variables that have the greatest influence on the projection as revealed from the developed OPLS-DA model. (**D)** Permutation test (n = 200) as a validation criterion for the OPLS-DA model, the test verifies the validity of the model with negative intercept for the Q2 values.

OPLS-DA was likewise employed as supervised model to identify showing likewise complete segregation of the authentic samples away from the commercial preparation with a fitness of data R2(X) of 70.6%, R2(Y) of 99.7% and predictive ability Q2 of 97.1% (Fig. 6A). The volatiles responsible for such clustering can be unraveled for the corresponding S-plot (Fig. 6B) and VIP plot (Fig. 6C) where peaks related to benzaldehyde, *endo*-borneol, terpinen-4-ol, cinnamaldehyde and cinnamyl alcohol were found to be more enriched in the authentic samples, while peaks of nonanoic acid, vanillin, alletone/furaneol and maltol were related to commercial products in agreement with (Table 2). Regarding the validity of OPLS model, permutation test with negative Q2 intercept value (Fig. 6D) and CV-ANOVA with *p* < 0.05 confirmed for its significance and no overfit. Interestingly, univariate analysis also showed comparable results with OLPS-DA model as revealed from the volcano plots (Fig. S4) where peaks related to *endo*-borneol, terpinen-4-ol and cinnamaldehyde were found enriched in the authentic samples. On the other hand, peaks related to benzyl alcohol was found significant and more enriched in the Naturelle^®^ preparation while peaks of vanillin, alletone/furaneol, methyl octanoate and maltol were related to Nu-prep LELAKI^®^ preparation posing these volatiles as differential metabolites for tongkat ali aroma profile.

## Conclusion

A comparative multiplexed metabolomics approach of NMR and GC–MS based profiling has been developed for the authentication, profiling, and standardization of tongkat ali compared to its commercial products. A total of 15 metabolites have been identified using NMR dataset mostly ascribed to sugars, organic and fatty acids, in addition to quassinoids as cinnamates as secondary metabolites. The multivariate analysis of the NMR fingerprint revealed for differences between the authentic and the commercial products with peaks related to fatty acids and 13,21-dihydroeurycomanone found to be more enriched in the authentic samples compared to commercial products. Asides, volatile profile of tongkat ali and two of its commercial products were characterized via SPME GC–MS analysis. The profile of the authentic root was relatively more similar to one commercial product Naturelle^®^ than Nu-prep LELAKI^®^ and indicating a potential contribution from the industrial processing and pharmaceutical formulation. The three products differed regarding the most abundant class, while the authentic product was rich in cinnamaldehyde and β-caryophyllene, Nu-prep LELAKI^®^ was abundant in methyl octanoate (fatty acid/ester). Markers for processing and thermal treatment was suggested based on the detection of maltol and 2-acetylpyrroleThe current research might help in authentication of tongkat ali as major flavor component in food, i.e., coffee in addition to nutraceuticals based on such multi-analytical platforms. Application of other metabolomics tools such as liquid chromatography mass spectrometry should provide better insight on secondary metabolome composition and impact of formulation in that major drug.

## Supplementary Information

Supplementary Information.

## Data Availability

The data presented in this study are available on request from the corresponding author.
