# Supplementary material for: Integrated comparative metabolite profiling via NMR and GC–MS analyses for tongkat ali (Eurycoma longifolia) fingerprinting and quality control analysis
Source: Sci Rep. 2023 Feb 13;13:2533. doi: 10.1038/s41598-023-28551-x (PMC9925447; doi:10.1038/s41598-023-28551-x)
Supplement: Supplementary file 1 — Supplementary Information. [file 41598_2023_28551_MOESM1_ESM.docx]

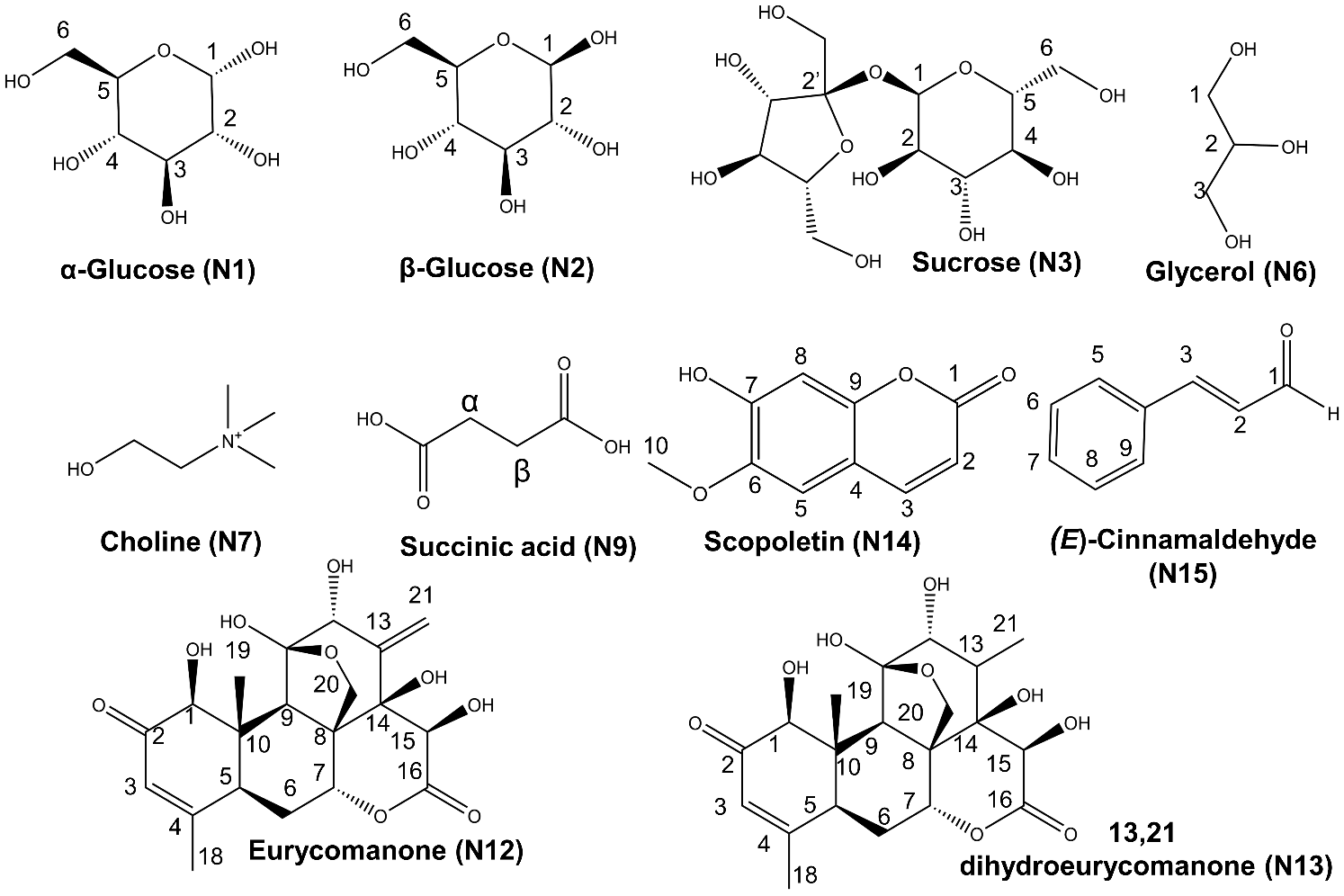


**Fig. S1:** Structure of the major primary and secondary metabolites detected in *E. longifolia* root extract. Carbon numbering system for each compound is based on analogy rather than IUPAC rules. Metabolite numbers follow those listed in **Table S1** for metabolite identification using 1D- and 2D-NMR.


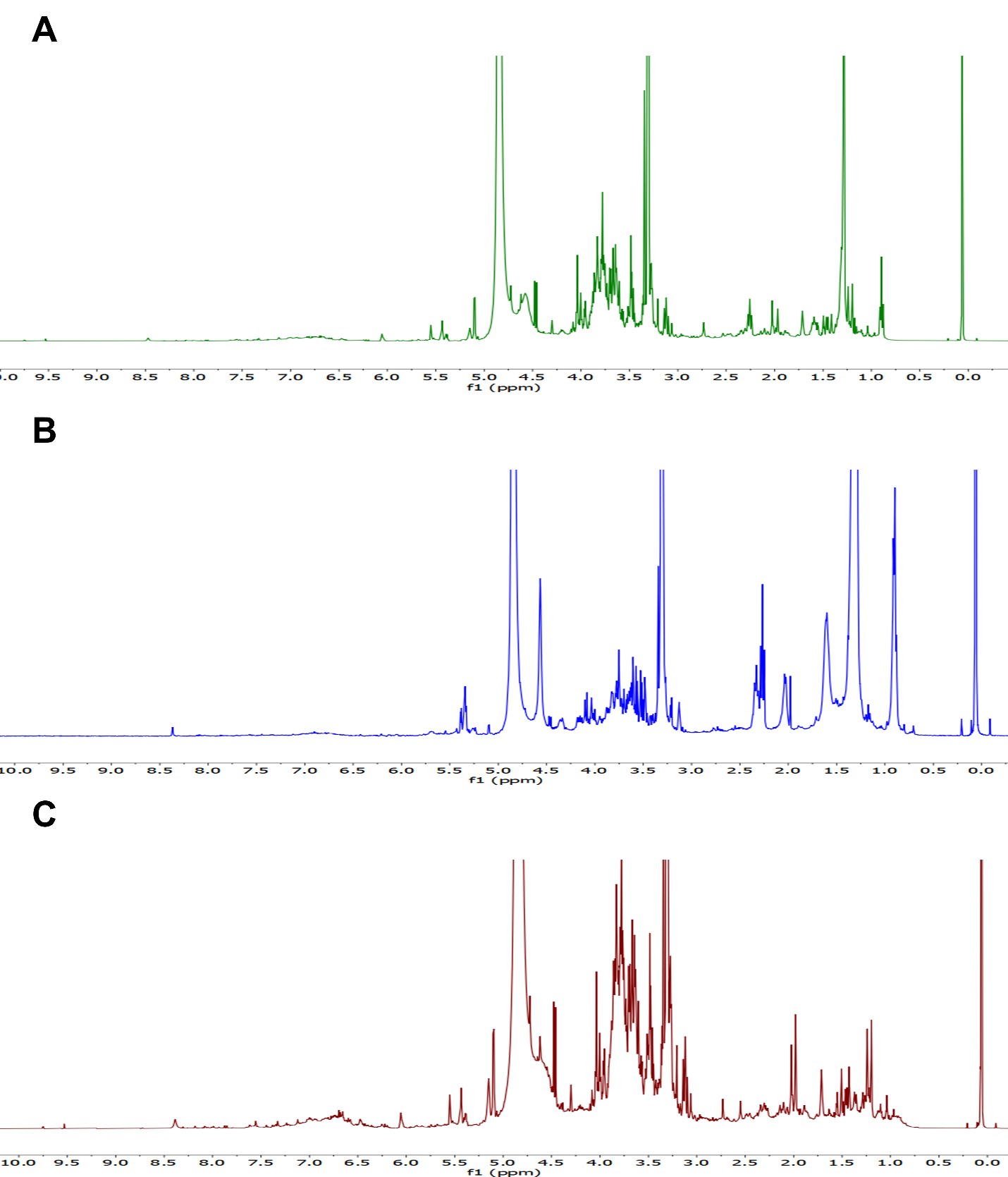


**Fig. S2**: Representative ^1^H NMR spectra of **(A)** Naturelle^®^, **(B)** Long jack plus^®^, and **(C)** Nu-prep LELAKI^®^ commercial preparations**.**


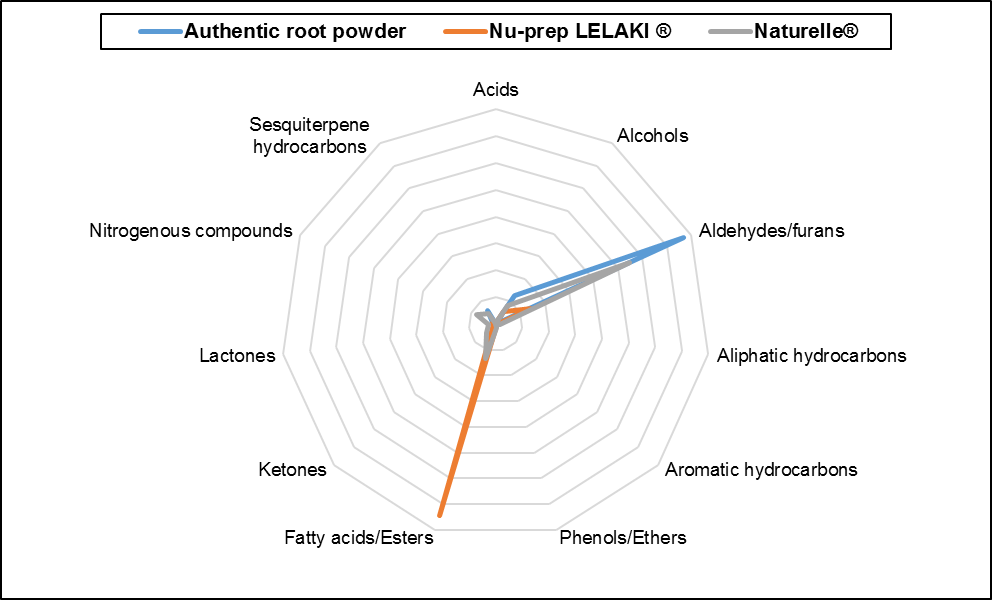


**Fig. S3**: Radar plot showing relative abundance (%) for different chemical classes of volatiles in authentic tongkat ali root powder, Nu-prep LELAKI ^®^, and Naturelle^®^ products as analyzed by SPME coupled with GC/MS


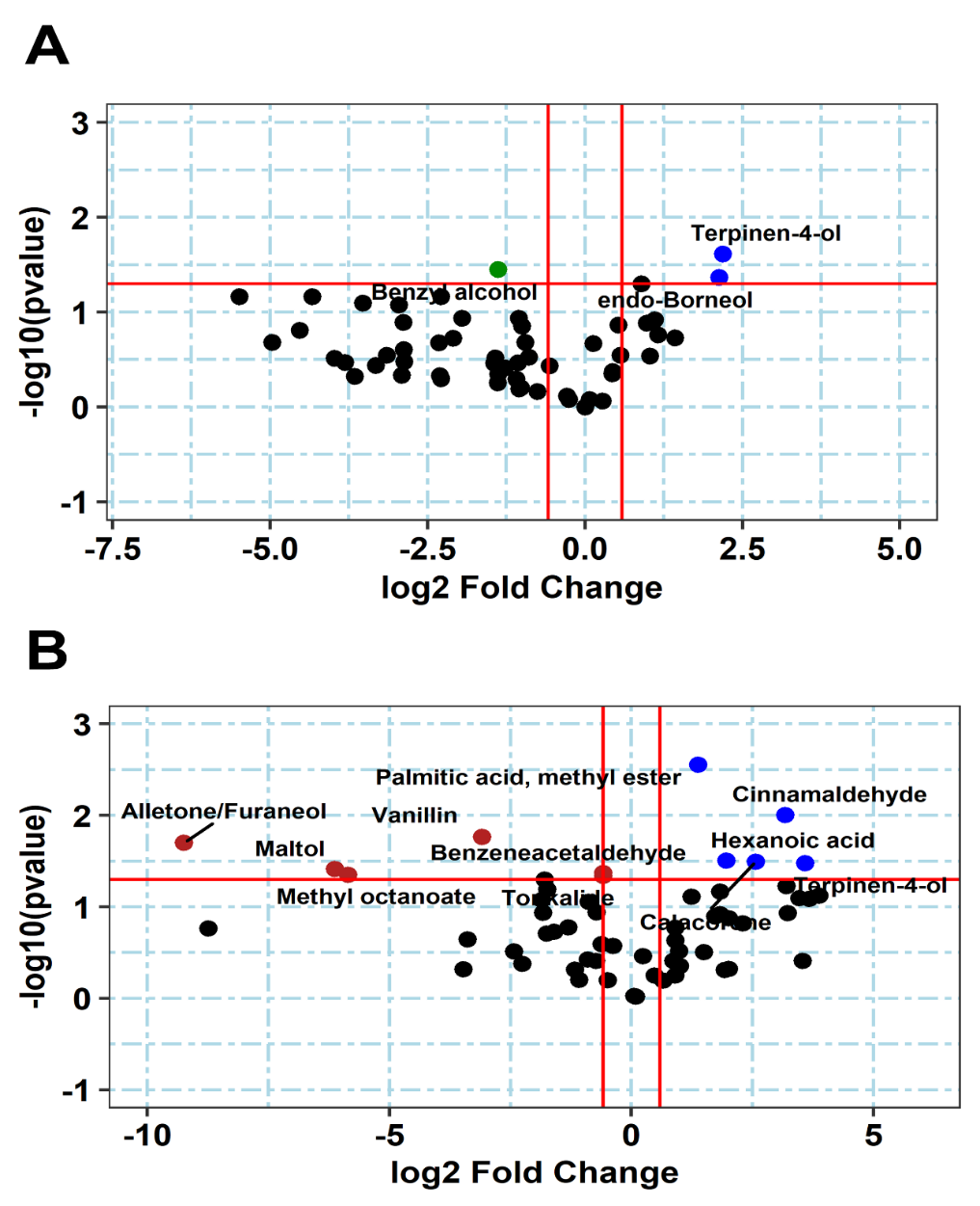


**Fig. S4**: Volcano plots of the GC-MS metabolomics dataset for (A) authentic tongkat ali vs Naturelle^®^ samples and (B) authentic tongkat ali vs Nu-prep LELAKI^®^ samples. The x-axis is the mean ratio fold-change (plotted on a log 2 scale) of the relative abundance of each metabolite. The y-axis is statistical significance reported as -log10 (*p*-value). Blue dots refer to increased metabolites in the authentic root samples. Pink and Green dots refer to statistically significant increased metabolites in Naturelle^®^ and Nu-prep LELAKI^®^ samples, respectively.

**Table S1:** ^1^H-NMR quantification of most primary and secondary metabolites detected in different samples of tongkat ali (*E. longifolia*). Values are expressed as μg/mg extract ± S.D (*n* = 3). Chemical shifts used for metabolite quantification were determined in methanol-*d*_4_ and expressed as relative values to HMDS (0.94 mM final concentration). Statistical analysis is carried out by one-way analysis of variance (ANOVA) followed by Dunnett's test for multiple group comparison of the commercial against the authentic samples with significance value at *p* ≤ 0.05.

| **Metabolite** | **Protons used in quantification** | **Authentic samples** | **Naturelle ^®^** | **Long jack plus ^®^** | **Nu-prep LELAKI ^®^** |
| --- | --- | --- | --- | --- | --- |
| *α*-Glucose **(N1)** | H-1 | 2.796 ± 0.280 | 7.863 ± 0.520 * | 1.101 ± 0.965 * | 38.000 ± 0.122 * |
| *β*-Glucose **(N2)** | H-1 | 4.287 ± 0.352 | 14.928 ± 0.177 * | 3.818 ± 0.776 | 48.922 ± 0.077 * |
| Sucrose **(N3)** | H-1 | 14.037 ± 0.406 | 10.000 ± 0.115 * | 5.730 ± 1.358 * | 11.365 ± 0.401 * |
| *α*-Fructofuranose **(N4)** | H-5 | 10.603 ± 0.067 | 18.194 ± 0.764 * | 6.103 ± 0.243 * | 17.881 ± 0.121 * |
| *β*-Fructofuranose **(N5)** | H-3 | 3.884 ± 0.148 | 28.287 ± 0.412 * | 1.479 ± 0.087 * | 15.136 ± 0.060 * |
| Glycerol **(N6)** | H-1 | 1.509 ± 0.001 | 5.015 ± 0.207 * | 1.017 ± 0.050 * | 5.642 ± 0.030 * |
| Acetic acid **(N8)** | H-2 | 0.200 ± 0.032 | 4.588 ± 0.172 * | 0.091 ± 0.009 | 0.676 ± 0.001 * |
| Eurycomanone **(N12)** | H-1 | 3.354 ± 0.308 | 37.004 ± 1.528* | 2.257 ± 0.791 | 18.865 ± 0.137* |
| Scopoletin **(N14)** | H-2 | 4.724 ± 0.341 | 0.212 ± 0.086 * | 0.106 ± 0.184 * | 1.070 ± 0.054 * |
| *(E*)-Cinnamaldehyde **(N15)** | H-1 | 1.906 ± 0.301 | nd | 0.052 ± 0.052 * | 0.021 ± 0.001 * |

* Significant metabolite with p ≤ 0.05

nd, not detected
